# Corrosion Performance and Post-Corrosion Evolution of Tensile Behaviors in Rebar Reinforced Ultra-High Performance Concrete

**DOI:** 10.3390/ma18112661

**Published:** 2025-06-05

**Authors:** Yuchen Zhang, Sumei Zhang, Xianzhi Luo, Chaofan Wang

**Affiliations:** 1 School of Intelligent Civil and Ocean Engineering, Harbin Institute of Technology, Shenzhen, University Town, Shenzhen 518055, China; yczhang_hit@163.com (Y.Z.); luoxianzhi_hit@163.com (X.L.); cfwang_hit@163.com (C.W.); 2 Guangdong Provincial Key Laboratory of Intelligent and Resilient Structures for Civil Engineering, Harbin Institute of Technology, Shenzhen, Shezhen 518055, China

**Keywords:** ultra-high-performance concrete (UHPC), corrosion cracking pattern, steel fiber, rebar, direct tensile test

## Abstract

The application of rebar reinforced ultra-high-performance concrete (R-UHPC) has been increasingly adopted in engineering structures due to its exceptional mechanical performance and durability characteristics. Nevertheless, when subjected to combined saline and stray current conditions, R-UHPC remains vulnerable to severe corrosion degradation. This investigation examined the corrosion performance and tensile behavior evolution of R-UHPC containing 2.0 vol% copper-coated steel fiber content and HRB400 steel rebar with a reinforcement ratio of 3.1%. The accelerated corrosion process was induced through an impressed current method, followed by direct tensile tests at varying exposure periods. The findings revealed that the embedding of rebar in UHPC led to the formation of fiber-to-rebar (F-R) conductive pathways, generating radial cracks besides laminar cracks. The bonding between rebar and UHPC degraded as corrosion progressed, leading to the loss of characteristic multiple-cracking behavior of R-UHPC in tension. Meanwhile, R-UHPC load-bearing capacity, transitioning from gradual to accelerated deterioration phases with prolonged corrosion, aligns with steel fibers temporally. During the initial 4 days of corrosion, the specimens displayed surface-level corrosion features with negligible steel fiber loss, showing less than 4.0% reduction in ultimate bearing capacity. At 8 days of corrosion, the steel fiber decreased by 22.6%, accompanied by an 18.3% reduction in bearing capacity. By 16 days of corrosion, the steel fiber loss reached 41.5%, with a corresponding bearing capacity reduction of 29.1%. During the corrosion process, corrosion cracks and load-bearing degradation in R-UHPC could be indicated by the ultrasonic damage factor.

## 1. Introduction

Ultra-high-performance concrete (UHPC), designed according to the optimal packing theory, is an advanced cementitious material characterized by ultra-high strength, enhanced toughness, and superior durability [1,2,3]. Its uniform, dense microstructure and extremely low porosity grant UHPC remarkable impermeability, carbonation resistance, and freeze–thaw durability compared to ordinary concrete [4,5,6,7]. The addition of steel fibers enhances the deformation and bearing capacity of UHPC, while their strong bonding with the matrix notably enhances the crack resistance of specimens [8,9,10,11]. At a typical steel fiber content of 2.0%, UHPC demonstrates over 20% strength increase relative to plain UHPC, and the maximum crack width remains below 0.2 mm when the tensile strain reaches 0.35% [12]. The superior mechanical properties and crack resistance of UHPC are essential for improving the functional performance and durability of engineering structures [13,14,15].

For structural applications, steel rebar is typically embedded within UHPC to form rebar-reinforced UHPC (R-UHPC), effectively meeting both load-bearing demands and crack width requirements [16,17,18,19]. Experimental investigations of UHPC-NC composite beams conducted by Ji et al. [20,21] revealed that the embedding of steel rebar in the UHPC layer enhanced the ultimate shear capacity by 28.6% relative to non-reinforced counterparts. Furthermore, the application of R-UHPC in tension zones of structures effectively limits crack propagation and controls the maximum crack width. Luo et al. [12] demonstrated that R-UHPC with 2.0% steel fibers and 3.0% rebar ratio not only maintained maximum crack width below 0.10 mm at rebar yielding but also achieved 62% greater strain capacity at 0.20 mm crack width compared to UHPC specimens with identical fiber content. However, when exposed to corrosive environments, the electrochemical corrosion risk of R-UHPC may be exacerbated by the presence of steel fibers and corrosive medium penetration.

Existing research on R-UHPC predominantly investigates cracking and mechanical behavior under conventional service conditions [22,23,24,25], creating a critical research gap given that material degradation in severe corrosive environments can undermine the load-bearing capacity of R-UHPC structures. This gap is particularly alarming considering that UHPC corrosion studies indicate that chloride ion penetration and sulfate attack reduce the strength of UHPC specimens [26,27,28,29,30,31]. Song et al. [32] demonstrated strength reduction in UHPC after 180-day exposure to marine environments: high-temperature seawater, wet–dry cycles, and immersion, with the most severe degradation occurring in high-temperature seawater, and the compressive strength of the corroded UHPC with 3.0% fiber content decreased by more than 10%. Similarly, Yang et al. [33] tested the compressive strength of UHPC exposed to sulfate environments for 540 days and found that the compressive strength of UHPC decreased gradually with increasing concentrations of sulfate ions. Crucially, these findings exclusively address UHPC, leaving the mechanical behavior of R-UHPC in high-salinity environments requiring further investigation.

The conductive network formed by steel fibers reduces the electrical resistivity of UHPC, thereby accelerating its corrosion process in electrochemical corrosion environments [34,35,36,37]. Experimental investigations by Song et al. [38] under constant 60 V accelerated corrosion for 28 days revealed that higher fiber contents lead to a reduction in the electrochemical resistance of UHPC, with the current for the 3% fiber content UHPC being roughly 50 times that of specimens with 1.0% fiber content. Han et al. [39] performed 55.5-h electrical accelerated corrosion tests on UHPC containing 1.0–3.0% steel fibers, finding that steel fiber network connectivity governs corrosion-induced cracking susceptibility and revealing specimens with 1.0% fibers exhibited negligible corrosion, compared to 9.75% and 14.54% volumetric losses in 2.0% and 3.0% fiber specimens. CT-based analysis by Lv et al. [40] on 2.0% fiber-content UHPC demonstrated edge-localized laminar cracking after 10-day corrosion, with cracking severity correlating strongly with fiber distribution density. In this context, the embedding of rebar in UHPC may introduce additional conductive networks, making R-UHPC more vulnerable to corrosion in high-salinity and stray current environments. However, current research has predominantly focused on the electrochemical corrosion process and conductive network formation in plain UHPC at the material scale, leaving the corrosion performance of R-UHPC largely unexplored. The influence of rebar on the generation of internal conductive pathways and its impact on the electrochemical corrosion cracking pattern of R-UHPC remains to be further investigated.

The objective of this study is to evaluate the conductive pathways and corrosion cracking pattern of R-UHPC, assess its tensile crack resistance and load-bearing capacity after corrosion, and reveal the potential relationship between the corrosion performance and tensile behavior of R-UHPC. To achieve this, the experimental program examined R-UHPC containing 2.0% steel fibers and 3.1% rebar ratio through accelerated corrosion tests spanning six periods (0, 2, 4, 6, 8, and 16 days) followed by direct tensile tests. The width of corrosion-induced surface cracks and the number of steel fibers in the cross-section of corroded specimens were quantitatively assessed. Additionally, ultrasonic nondestructive testing (UNDT) was conducted to monitor the internal cracking evolution, and Digital Image Correlation (DIC) was employed to measure full-field strain characterization of the specimens. By analyzing the corrosion morphology, crack development, and constituent degradation of steel fiber and rebar, the corrosion patterns of R-UHPC were summarized. Furthermore, the effects of corrosion on the failure modes, load-bearing capacity, and deformation capacity of R-UHPC were explored, focusing on tensile crack propagation, peak load, and ductility.

## 2. Materials and Methods

### 2.1. Material Property

The UHPC material used in this study was a commercially available product provided by Hongri Tenacal New Material Technology Co., Ltd. (Zhejiang, China), consisting of pre-mixed powder, superplasticizer, and copper-coated steel fibers at a volume content of 2.0%, a proportion commonly adopted in the current research and application of UHPC [14,40,41,42]. The raw materials for UHPC conformed to the Chinese standard T/CCPA 20-2020 Premix of Ultra-High-Performance Concrete [43], which specifies rigorous quality control requirements for material composition. According to current research on the effects of fiber on UHPC, the use of hybrid fibers can meet the requirements for both workability and ultimate strength [44,45]. The copper-coated steel fibers consisted of half straight fibers with 0.20 mm diameter × 16 mm length and half end-hooked fibers with 0.22 mm diameter × 16 mm length. The core of the fiber is carbon steel with a nominal ultimate tensile strength of 2800 MPa, coated with a uniform copper layer. The mechanical properties of the UHPC were tested according to the Chinese standards GB/T 31387-2015 Reactive powder concrete [46] and T-CBMF 37-2018 Ultra-high-performance concrete-Basic properties and test methods [47], while the steel rebars were tested according to the international standard GB/T 228.1-2010 Metallic materials-Tensile testing-Part 1: Method of test at room temperature [48]. The test results are detailed in Table 1 and Table 2.

### 2.2. Specimen Preparation

**Figure 1:**
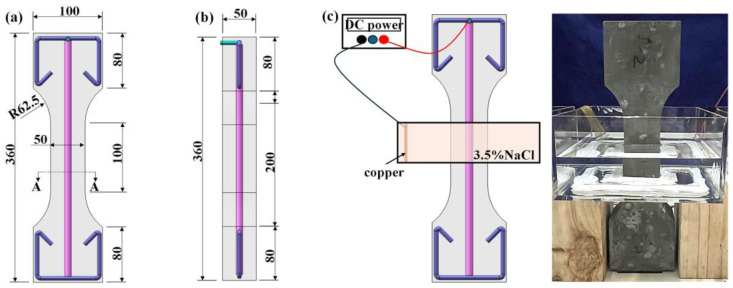
R-UHPC specimen sizes: (**a**) front view; (**b**) side view; and (**c**) corrosion setup.

Current research on corrosion performance or tensile behavior of UHPC and R-UHPC primarily employs prismatic and dog-bone configurations [49,50,51]. The dog-bone specimen design, characterized by arc transition regions and enlarged gripping ends relative to prismatic counterparts, has demonstrated superior effectiveness in preventing end failure during tensile testing. In this investigation, dog-bone specimens were selected to evaluate the post-corrosion tensile performance of R-UHPC. All specimens shared identical overall dimensions of 50 × 100 × 360 mm with a tensile segment of 50 × 50 × 100 mm, as illustrated in Figure 1. Each specimen incorporated a single centrally positioned HRB400 rebar with a nominal diameter of 10 mm. The R-UHPC specimens’ thickness was set to 50 mm with a 20 mm nominal concrete cover of rebar, following typical specifications for structural strengthening applications [19,20]. Triplicate specimens containing 2.0% steel fibers and 3.1% rebar ratio were fabricated for each parameter combination (Table 3) to account for potential casting-induced variability.

**Figure 2:**
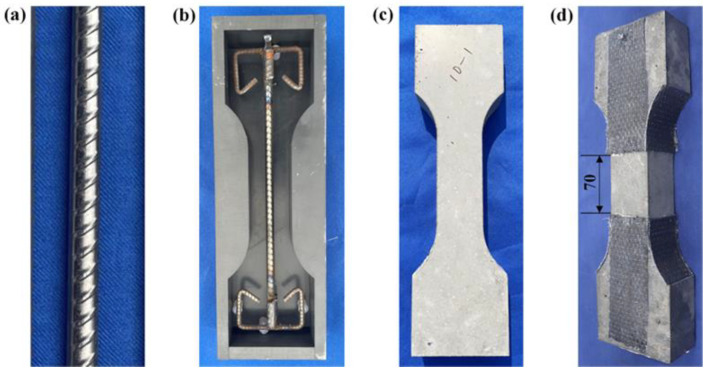
R-UHPC specimen preparation: (**a**) rebars cleaning; (**b**) rebar cage positioning; (**c**) cured specimen; and (**d**) arch zones strengthening.

The fabrication process of R-UHPC specimens is illustrated in Figure 2. Prior to concrete placement, all rebars were cleaned to remove oil residues, dirt, and other surface contaminants. This pretreatment ensured a uniformly clean surface, guaranteeing that subsequent corrosion observed in the rebar originated exclusively from the controlled electrochemical process, thereby enabling evaluation and quantitative analysis of corrosion-induced damage. The mixing and curing processes adhered to the Chinese standard T/CCPA 23-2021 Recommendations for on-site placing ultra-high-performance concrete [52], ensuring standardized specimen preparation. After 24 h of casting, the specimens were demolded and then cured in a standard controlled chamber maintained at 20 ± 2 °C with relative humidity ≥ 95% for 28 days. Prior to testing, the arc transition regions of the specimens were externally reinforced with unidirectional carbon fiber-reinforced polymer (CFRP) sheets to prevent stress concentration-induced failure, and the measuring section length of the specimen was 70 mm (Figure 2d).

### 2.3. Electrochemical Corrosion Test

In UHPC corrosion research, common accelerated corrosion methods include saltwater immersion, wet–dry cycling, immersion with prefabricated cracks, and electrochemical corrosion [29,35,39,49]. Among these, the electrochemical corrosion method significantly speeds up the corrosion process and has proved excellent efficacy in studies of corrosion mechanisms and post-corrosion performance degradation. Therefore, electrochemical corrosion was induced by direct current application to the embedded rebar within R-UHPC specimens. Carbonation alters the pore structure and size distribution of cementitious matrices, thereby enhancing chloride penetration rates and reducing material alkalinity [53,54,55,56], thereby accelerating the corrosion of R-UHPC. Existing studies demonstrate that ordinary concrete exposed to natural atmospheric environment for 1 year develops carbonation depths of merely 2.0 mm [57]. Compared to ordinary concrete, the UHPC high-density microstructure and exceptionally low permeability further inhibit carbonation progression in R-UHPC specimens [58]. In this study, since accelerated corrosion testing was limited to only 16 days, the carbonation effects may be minimal. Consequently, carbonation was excluded from this study. In long-term exposure conditions, carbonation may become more pronounced, warranting attention in future R-UHPC durability research.

The R-UHPC functioned as the anode with the rebar connected to the positive terminal of a DC power supply via conductive wires in the corrosion setup. Simultaneously, a copper mesh served as the cathode, immersed in a 3.5 wt% NaCl electrolyte solution (Figure 1c). The DC power supply operated within a voltage range of 0–305 V, and a constant corrosion current of 40 mA was applied to give corrosion periods of 0 (control, no power), 2, 4, 6, 8, or 16 days (Table 3). The central 50 mm segment was chosen to realize targeted corrosion, concentrating electrochemical reactions in the principal tensile region.

**Figure 3:**
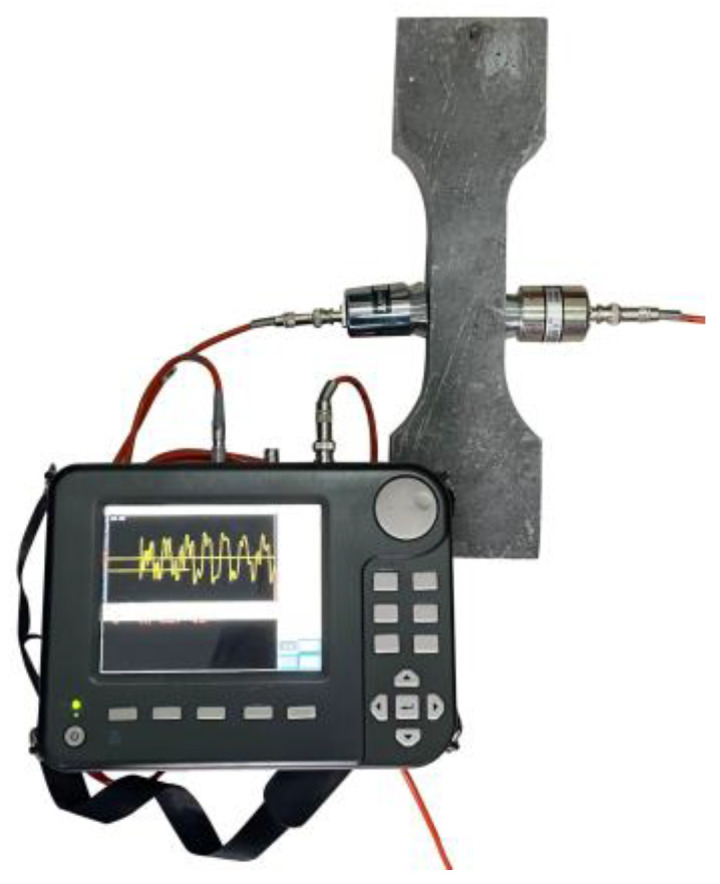
Ultrasonic nondestructive test.

With progressive electrochemical corrosion, the severity of corrosion damage in R-UHPC specimens increased gradually, accompanied by continuous widening of surface corrosion cracks. To quantify this surface deterioration, the widths of the visible cracks were measured using a crack width gauge with a resolution of 0.01 mm. Meanwhile, internal corrosion damage was evaluated through ultrasonic nondestructive testing (UNDT) (Figure 3), which analyzed changes in ultrasonic wave transit time and velocity in R-UHPC before and after corrosion, enabling the quantification of cumulative internal corrosion damage in specimens. Notably, the reliability of UNDT has been extensively proved in freeze–thaw cycling and corrosion studies of both conventional concrete and UHPC, and the damage factor *D*, derived from ultrasonic parameters, is widely used to quantify internal damage and material performance degradation [59,60,61]. In accordance with this established methodology, this study employed the damage factor *D* (Equation (1)) to characterize the corrosion damage in R-UHPC specimens at different corrosion periods, where *E*_0_, *V*_0_, and *T*_0_ denote the modulus of elasticity, ultrasonic velocity, and ultrasonic transit time of the uncorroded R-UHPC, respectively, whereas *E*_t_, *V*_t_, and *T*_t_ represent these parameters following corrosion.

*D* = 1 − *E*_t_/*E*_0_ = 1 − (*V*_t_/*V*_0_)^2^ = 1 − (*T*_0_/*T*_t_)^2^ (1)

### 2.4. Direct Tensile Test

**Figure 4:**
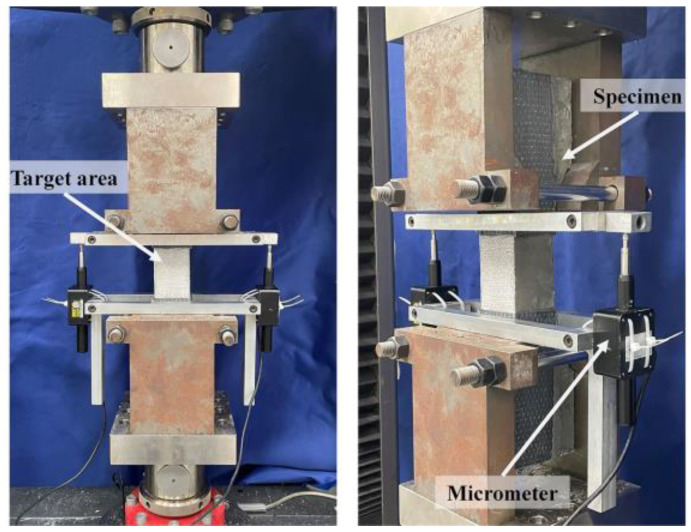
Direct tensile test setup.

Tensile tests were performed on corroded R-UHPC specimens in an electromechanical universal testing machine with a capacity of 100 kN (Figure 4). Prior to tensile loading, a pre-load of 5 kN was applied to each specimen to ensure proper alignment and centering. Subsequently, the tensile test was conducted under displacement control at a constant rate of 0.1 mm/min, which was considered adequate to capture the tensile mechanical response and the crack development process of R-UHPC under static conditions [62]. For precise displacement measurement, two micrometers were mounted on opposite sides of the target area to measure displacement, while the DIC system was employed to fully document the crack development during the tensile loading. Through this dual measurement approach, which synchronized acquisition of global displacement data (via micrometers) and localized strain field evolution (via DIC), enabled detailed characterization of the tensile damage progression in corroded specimens.

## 3. Conductive Corrosion Pathways

### 3.1. Fiber-to-Fiber Pathways and Fiber-to-Rebar Pathways

**Figure 5:**
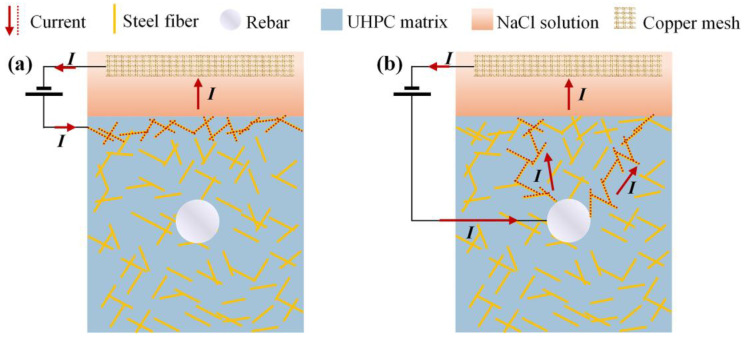
(**a**) F-F pathways and (**b**) F-R pathways.

Existing studies have confirmed the conductive networks formed by steel fibers within UHPC through CT scan analysis, with interconnections exceeding 80% of the total steel fiber content in specimens containing more than 2.0% steel fiber [39]. This phenomenon is attributed to the increased fiber content and enhanced connectivity facilitate the diffusion of corrosion current and the concentration of local current density, significantly promoting rapid current transmission within UHPC. As illustrated in Figure 5a, the current transport pathways within the fiber-to-fiber (F-F) pathways of R-UHPC under electrochemical corrosion conditions. Moreover, due to the exceptional impermeability of the UHPC matrix, the corrosive solution penetrates only the surface layer in early corrosion phases, resulting in preferential corrosion of steel fibers located near the specimen surface and exposed to the corrosive solution.

**Figure 6:**
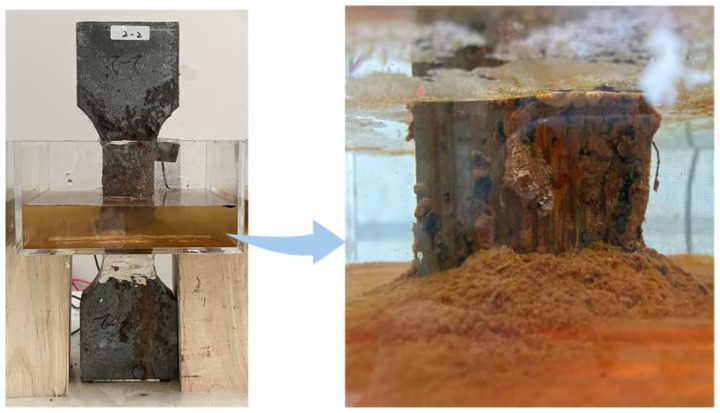
Fe(OH)_3_ produced by corrosion of R-UHPC.

After the incorporation of rebar within UHPC, corrosion current propagates not only through the F-F pathways but also via the fiber-to-rebar (F-R) conductive pathways (Figure 5), where current transmission within R-UHPC preferentially follows low-resistance pathways analogous to the behavior of parallel circuits, where current flows predominantly through lower-resistance branches. These F-R pathways lead to local current density elevation and expedite the penetration of corrosive medium into specimen, thereby accelerating corrosion of steel fibers in specific regions. Under the electrochemical environment, both the steel fibers and rebar act as the anode, where the dominant reaction is iron oxidation: Fe − 2e^−^→ Fe^2+^. Subsequently, the Fe^2+^ ions are unstable and are rapidly oxidized to Fe^3+^: 4Fe^2+^ + O_2_ + 6H_2_O → 4Fe(OH)_3_↓. Under the combined effects of diffusion and electric fields, the reddish-brown Fe(OH)_3_ accumulates in the solution (Figure 6). The corrosion process involves simultaneous oxygen reduction reactions: O_2_ + 2H_2_O + 4e^−^ → 4OH^−^ [63,64,65]. Under oxygen-depleted conditions, an alternative cathodic reaction takes place: 2H_2_O + 2e^−^ → 2OH^−^ + H_2_↑ [63,64].

### 3.2. Voltage Evolution Under Constant-Current Corrosion Environment

**Figure 7:**
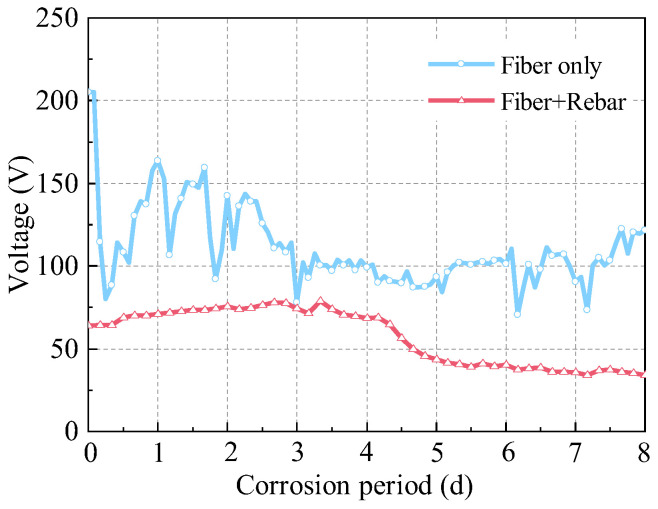
Voltage–corrosion period curves of R-UHPC.

Under constant-current corrosion conditions, the resistance of R-UHPC varies due to the corrosion of steel fibers along the conductive pathways and the development of microcracks in the matrix, leading to alterations in corrosion voltage. The experimental investigation compared the temporal evolution of voltage applied by the DC power supply over an 8-day corrosion period for three types of specimens (Figure 7): UHPC containing only 2.0% steel fibers without rebar, R-UHPC with only a 10 mm diameter rebar without fibers, and R-UHPC containing both 2.0% steel fibers and a 10 mm diameter rebar.

During the 8-day accelerated corrosion process, the R-UHPC reached the DC power supply maximum output voltage of 305 V, which limited the current to merely 5–6 mA, significantly below the target 40 mA. Consequently, this specimen voltage curve was not included in Figure 7 to ensure comparability of the remaining datasets. This inhibited corrosion activity resulted from the ultra-low porosity of the UHPC matrix, where corrosion current transmission was predominantly confined to ionic migration through highly restricted pore networks. The specimen’s electrical resistance remained governed by the intact UHPC matrix, resulting in no measurable voltage reduction during the 8-day corrosion period.

The voltage of UHPC containing only 2.0% steel fibers was consistently lower than that of R-UHPC with rebar only during the corrosion period, primarily due to the potential formation of conductive F-F pathways within the UHPC that substantially reduced the overall resistivity. Given that the electron conductivity of steel fibers is significantly higher than the ionic mobility of pore solutions, corrosion currents preferentially propagate through the fiber networks, significantly accelerating the corrosion process. Interestingly, the voltage of the UHPC exhibited fluctuations during the corrosion process, with initial voltages starting at 205.3 V, decreasing to 88.6 V within 8 h, and later increasing to 163.5 V after 16 h. This behavior may be attributed to two competing mechanisms: (1) steel fiber corrosion-induced microcracks in the UHPC matrix accelerated solution penetration, thereby reducing overall resistivity; (2) the rapid corrosion and dissolution of steel fibers along conductive paths subsequently increased resistivity. These concurrent phenomena cause the observed fluctuations in specimen resistivity and corresponding changes in the voltage.

Likewise, under the 40 mA constant current environment, the initial voltage for R-UHPC with both steel fibers and rebar was markedly lower at 63.9 V, approximately 60% lower than that of fiber-only specimen, suggesting that in the early stage of corrosion, the corrosion current flowed through paths involving both steel fibers and rebar, rather than through fibers alone. Throughout the 8-day corrosion period, the voltage of R-UHPC remains consistently lower than that of both fiber-only and rebar-only specimens. Within the first 3 days, the voltage in the specimen containing both fibers and rebar gradually increased, likely due to the slow dissolution of surface-near fibers reducing overall conductivity. Additionally, the rehydration of the UHPC matrix could further increase the voltage by enhancing the resistivity of the material. The drop in voltage after 4 days might be attributed to the corrosive solution penetration through UHPC matrix microcracks and pores, establishing continuous ionic transport paths to the rebar, thereby reducing the resistance of the specimen and leading to a gradual decline in the voltage.

## 4. Corrosion Performance

### 4.1. Corrosion Morphology and Crack Propagation

**Figure 8:**
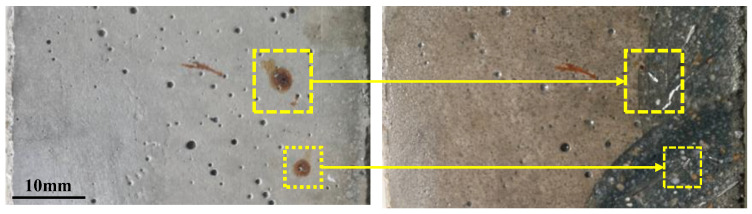
Steel fibers under the corrosion area.

**Figure 9:**
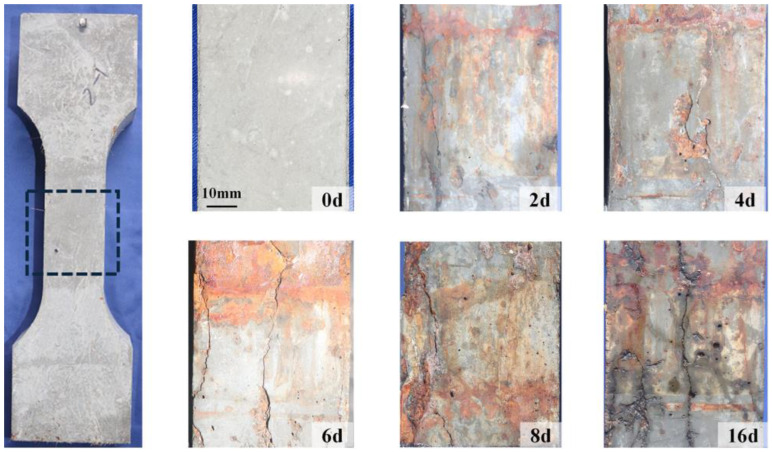
Surface morphology of R-UHPCs with different corrosion periods.

In the initial corrosion stages, reddish-brown corrosion products appeared on the R-UHPC surface. Upon grinding of selected corroded areas, underlying steel fibers were exposed (Figure 8), confirming preferential and rapid corrosion of surface-proximate steel fibers during the specimen corrosion. This phenomenon can be attributed to the synergistic combination of direct corrosive solution access and enhanced electrochemical activity at the conductive pathway. Following surface cleaning of corroded specimens, the morphology of the test sections is presented in Figure 9, where specimens representing different corrosion periods were distinct samples. Uncorroded specimens maintained smooth, homogeneous light gray surfaces, whereas corroded specimens exhibited widespread orange corrosion marks and developed corrosion-induced cracks, with the surface roughness and crack width increasing over time. After 6 days of corrosion, prominent longitudinal cracks appeared, mirroring longitudinal cracking documented in ordinary reinforced concrete members subjected to rebar corrosion [66,67]. This distinctive crack morphology suggested the potential occurrence of rebar corrosion in R-UHPC.

**Figure 10:**
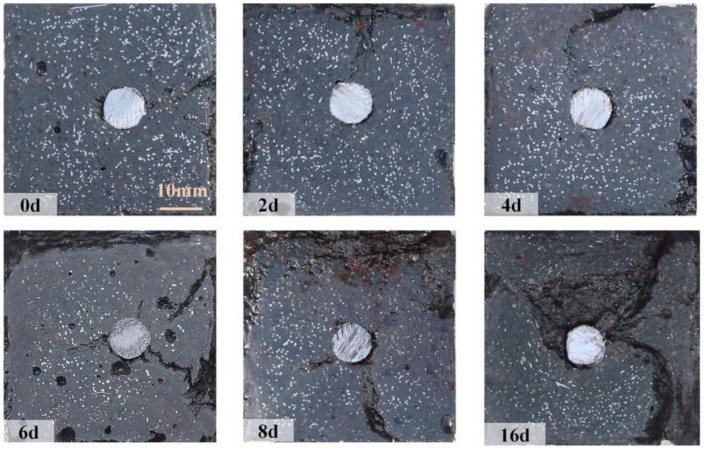
Cross-section morphology of R-UHPCs with different corrosion periods.

**Figure 11:**
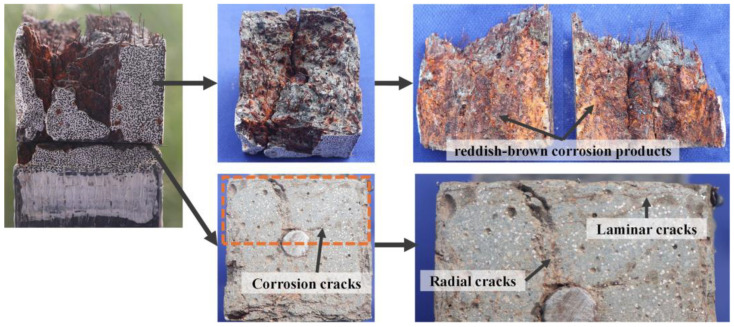
The corrosion signs in corrosion cracks.

As corrosion progressed, cracks propagated deeper into the UHPC matrix. Cross-sectional analysis of post-tension fractured specimens was conducted to investigate the internal crack development (Figure 10), using oil spray techniques to enhance the visualization of steel fiber distribution and crack paths. It should be noted that the analyzed cross-sections represent distinct specimens corresponding to different corrosion periods. In uncorroded specimens, minor cracks observed were exclusively attributed to stress concentrations in the UHPC matrix due to rebar deformation during tensile testing. In contrast, corrosion-induced cracks displayed distinct reddish-brown deposits (Figure 11), with their characteristic coloration serving as a direct indicator of electrochemical corrosion damage, providing clear differentiation from mechanically induced cracking.

Compared to the uncorroded R-UHPC, cross-sections of 2-day corroded specimens showed localized steel fiber corrosion at the edges, accompanied by the formation of laminar corrosion cracks parallel to the specimen. Notably, partially corroded steel fibers remained in these laminar cracks rather than being completely lost. The propagation of laminar cracks closely followed the direction of the F-F conductive pathways (Figure 5a), where steel fiber corrosion preferentially initiated, resulting in the stress concentration in the UHPC matrix that promoted the development of cracks. After 4 days of corrosion, the increased severity of steel fiber corrosion at the edges led to the expansion of the corrosion crack width. Furthermore, the F-R conductive pathways facilitated the emergence of radial cracks, which propagated inward from the specimen edges.

At 6 days, R-UHPC revealed distinct laminar cracks and radial cracks that aligned with conductive pathways (Figure 5). Within the 4 mm edge region, the majority of steel fibers had deteriorated, while continuous accumulation of low-density corrosion products from edge fiber corrosion promoted the initiation and development of cracks in the UHPC matrix. This process accelerated corrosive solution penetration and drove the progressive expansion of laminar cracking regions. Compared to 4-day corroded specimens, the cross-section of specimens corroded for 6 days showed fully developed radial cracks that connected external corrosive environments to internal rebar, accompanied by a significant reduction of steel fibers around these cracks. This observation indicated that F-R conductive pathways in specimens induced localized current concentration, which in turn accelerated electrochemical corrosion of fibers along these pathways and drove rapid crack propagation to the central rebar area. At the initiation regions of these radial cracks, steel fibers were almost entirely corroded away, and the surrounding UHPC matrix exhibited severe cracking and corrosion-induced spalling. This localized damage severity correlates with the high steel fiber density in these regions, as demonstrated in existing UHPC corrosion research [39].

After 8 days of corrosion, the R-UHPC specimens showed a noticeable expansion in the region of laminar corrosion cracks, with the maximum crack propagation depth reaching 9.2 mm from the section edge. Especially, the depth of laminar cracking displayed variability around the section, influenced by the random distribution and densities of steel fibers within the cross-section. Regions with greater fiber connectivity and density facilitated the establishment of continuous conductive pathways, which reduced electrochemical corrosion resistance and promoted deeper, more severe laminar cracks. As the corrosion process continued beyond 16 days, the severity of corrosion damage in the specimens intensified markedly, as evidenced by both increased width and quantity of radial cracks. In these regions of crack progression, steel fibers were nearly completely corroded away. Together, these findings demonstrate a clear correlation between conductive pathways and corrosion cracking patterns, confirming that F-F pathways and F-R pathways affect both the progression and severity of corrosion damage in R-UHPC.

### 4.2. Surface and Internal Corrosion Damage Development

**Figure 12:**
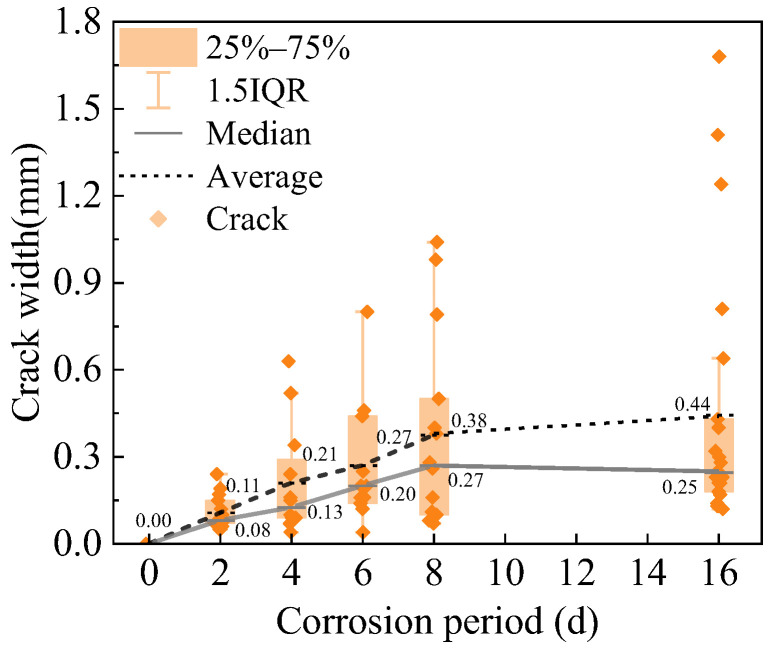
Surface crack width of R-UHPC.

Over the course of corrosion, the severity of surface corrosion damage gradually increased, accompanied by measurable growth in crack width. The experimental investigation quantitatively assessed the development of visible surface cracks, as shown in Figure 12, where each diamond-shaped marker represents an individual crack. Following 2, 4, 6, 8, and 16 days of electrochemical corrosion, the maximum crack widths, primarily longitudinal along the rebar direction, were 0.24, 0.63, 0.80, 1.04, and 1.68 mm, while the average crack widths were 0.11, 0.21, 0.27, 0.38, and 0.44 mm. The average crack width in R-UHPC exhibited a roughly linear increase over the first 8 days of corrosion, doubling from day 4 to day 8. This trend was driven by the continuous corrosion of steel fibers and rebar under constant-current conditions, which generated corrosion products that progressively widened the surface cracks.

**Figure 13:**
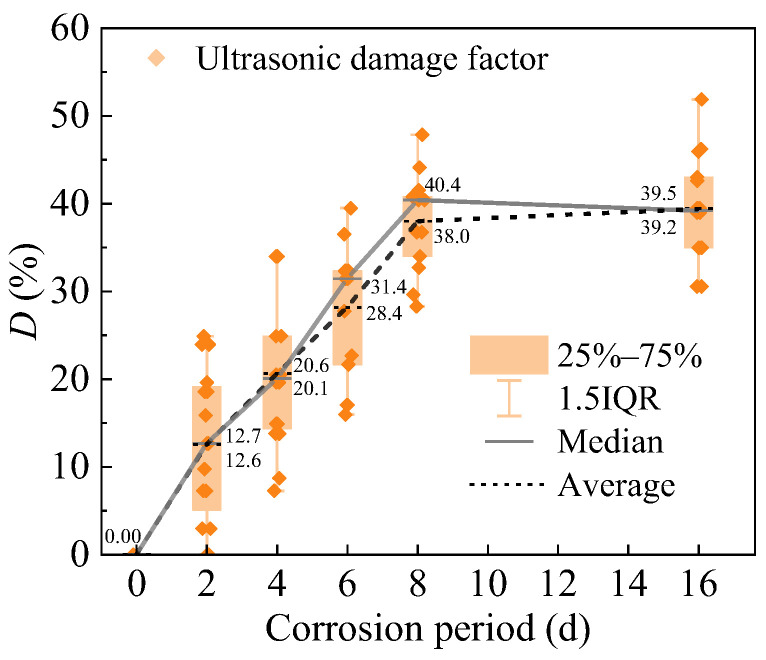
Ultrasonic damage factor of R-UHPC.

UNDT enables the detection of internal cracking conditions in R-UHPC, providing a quantitative assessment of internal cracking damage across varying corrosion periods through the ultrasonic damage factor (*D*). The UNDT results (Figure 13) revealed that the damage factor of R-UHPC specimens increased with corrosion period up to 8 days, after which it stabilized with no significant further changes. Interestingly, the variation in ultrasonic damage factors correlates well with the progression of surface crack widths over time, confirming its reliability as a corrosion damage indicator. Furthermore, both the average and median values of the damage factor demonstrated strong linear correlations with corrosion period. After 2, 4, 6, and 8 days of electrochemical corrosion, the average damage factors were 12.6%, 20.6%, 28.2%, and 38.0%, respectively. This progressive escalation reflects the cumulative effects of corrosion severity, manifested through increased density and width of internal cracks. At the 16-day stage of the accelerated corrosion test in R-UHPC, the average and median values of the ultrasonic damage factor were 39.5% and 39.2%, respectively, closely aligning with those recorded on day 8. This similarity may result from the severe internal cracking damage in the specimen after 8 days of corrosion, likely exceeding the UNDT detection limit due to critical cracking damage thresholds. Beyond this threshold, UNDT measurements of advanced corrosion damage become limited.

### 4.3. Steel Fiber Corrosion

**Figure 14:**
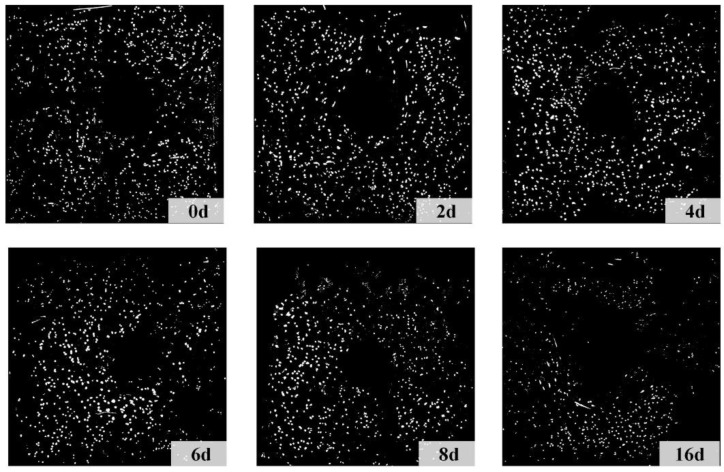
Steel fiber identification.

**Figure 15:**
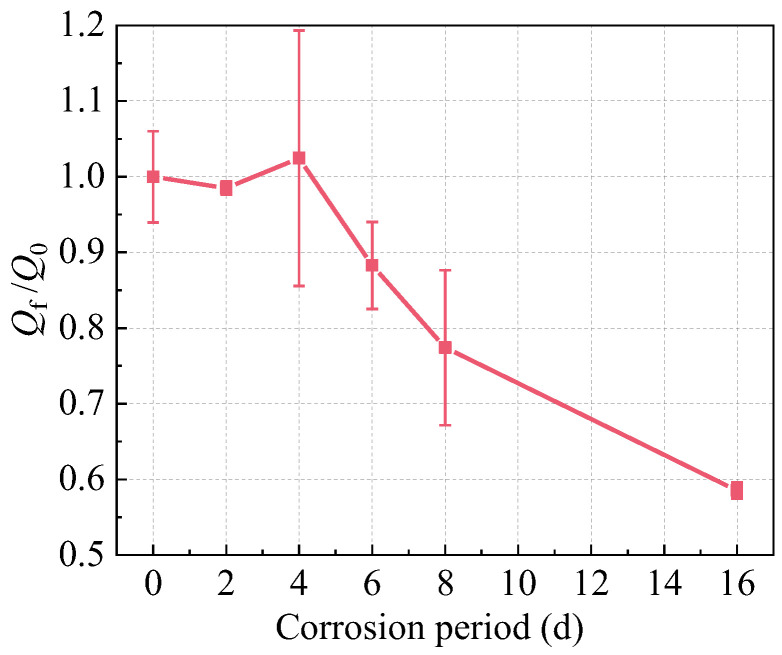
The effect of corrosion on the quantity of steel fibers in R-UHPC.

Corrosion progressively reduces the cross-sections or completely dissolves steel fibers within R-UHPC, resulting in a continuous decrease in steel fibers. The image recognition software ImageJ 2.14.0 was employed to identify steel fibers in corroded R-UHPC cross-sections (Figure 14). The relationship between the normalized steel fiber quantity (*Q*_f_/*Q*_0_) and corrosion period is presented in Figure 15, where *Q*_f_ represents the fiber quantity at a given corrosion period and *Q*_0_ denotes the initial fiber quantity of uncorroded R-UHPC.

During the initial 4 days of corrosion, R-UHPC cross-sections exhibited no significant reduction in average steel fiber quantity. Analysis of the steel fiber distribution (Figure 14) and the crack progression of R-UHPC sections (Figure 10) revealed that corrosion is predominantly confined within approximately 4 mm of the specimen surface in the initial corrosion stage, a phenomenon attributed to the limited penetration of corrosive solutions through the dense UHPC matrix. The corrosion patterns of affected fibers exhibited variation, showing complete dissolution to partial cross-sectional area reduction. Meanwhile, considering inherent variations in steel fiber distribution across different UHPC cross-sections, a small amount of localized fiber corrosion may not significantly affect the overall fiber quantity within a given section (Figure 15). These findings collectively demonstrate that R-UHPC can effectively preserve its structural integrity during initial corrosion periods, as the damage mechanism remains confined to surface regions and may not degrade the overall mechanical performance.

From day 4 to day 6 of corrosion, when complete radial cracks formed on the cross-sections, the majority of steel fibers adjacent to these cracks or within the 4 mm edge region of R-UHPC sections vanished by corrosion, resulting in a rapid 13.8% reduction in fiber quantity over only 2 days. By day 8 of corrosion, the fiber quantity had further decreased by 22.6% compared to uncorroded specimens. Even though the UHPC matrix has excellent permeability resistance, the corrosion-induced cracks accelerated the rapid penetration of the corrosive solution into the specimens, thus expediting the corrosion of internal steel fibers. However, after 8 days of corrosion, the rate of steel fiber reduction in R-UHPC exhibited a slight decrease. This phenomenon may be attributed to the expansion of radial cracks, which progressively increased rebar exposure to external corrosive solutions. Under constant-current conditions, the corrosion rate of the internal rebar may increase, thereby leading to a marginal decrease in the steel fiber corrosion rate. At 16 days, the quantity of steel fibers in the cross-section had decreased by 41.47% compared to uncorroded sections, primarily due to extensive corrosion of fibers surrounding the rebar.

### 4.4. Rebar Corrosion

**Figure 16:**
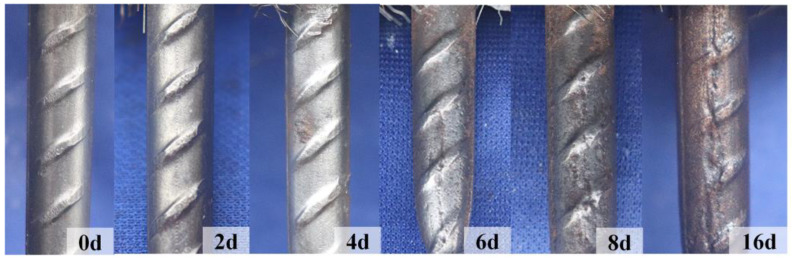
Rebar morphology of R-UHPC with different corrosion periods.

**Figure 17:**
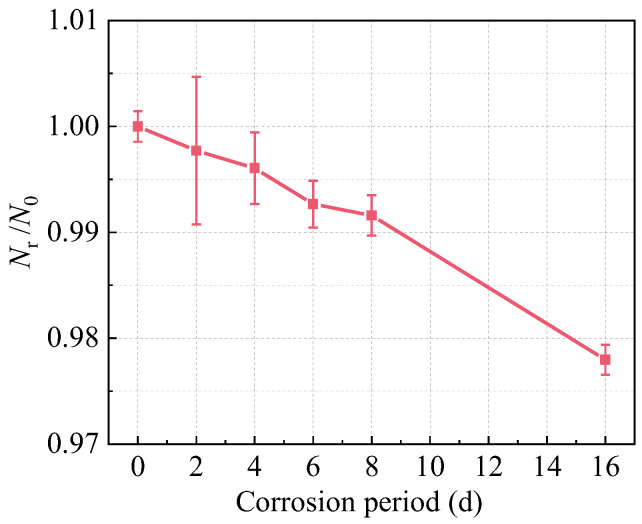
The effect of corrosion on rebar in R-UHPC.

**Figure 18:**
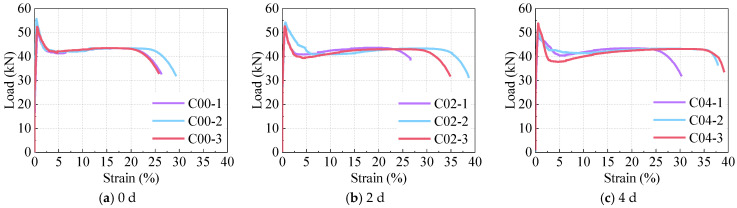

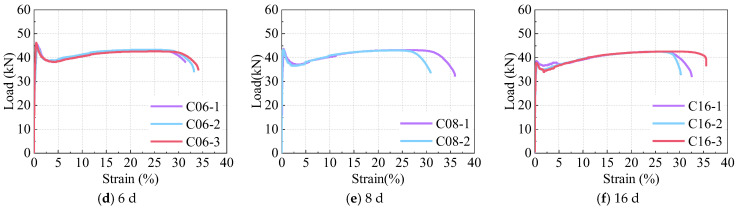
Load–displacement response.

The steel rebar embedded in R-UHPC undergoes progressive cross-sectional loss due to sustained corrosion. To characterize this degradation, the corrosion morphology (Figure 16) and severity (Figure 17) of rebar with 20 mm concrete cover were evaluated, with specimens sourced directly from R-UHPC that had completed tensile test, and the rebar entered strain-hardening after UHPC matrix fracture (Figure 18). For quantitative analysis of the corrosion-induced in rebar, the load-bearing capacity (*N*_r_) of corroded rebars was normalized against uncorroded reference values (*N*_0_), as plotted in Figure 17. Critically, since the degradation of load-bearing capacity primarily results from the corrosion-induced sectional loss of steel rebar, the normalized corrosion damage values (*N*_r_/*N*_0_) serve as a direct indicator for the actual cross-sectional area loss.

During the initial 2-day exposure period, mild surface roughening of the reinforcement was observed, with no pitting corrosion and 99.77% retention of the original cross-sectional area. Subsequently, by day 4, localized corrosion initiation became apparent through localized corrosion marks, corresponding to a 0.39% sectional loss and accompanied by the propagation of radial cracks toward the specimen interior. As exposure continued to 6 and 8 days, significant corrosion expansion occurred, characterized by multiple pits observed on the rebar due to the complete formation of radial cracks that enabled direct corrosive solution penetration. The corrosion rate of rebars in R-UHPC specimens slightly increased after 8 days, potentially due to significant radial crack damage that enhanced contact between the rebar and external corrosive medium. Ultimately, after 16 days, generalized corrosion occurred along the rebar because of multiple radial cracks, resulting in a 2.20% reduction in cross-sectional area. This transition from localized to generalized corrosion can be attributed to the following mechanism: the formation and expansion of radial cracks in the specimen cross-section established continuous channels for corrosive solution penetration, as evidenced by morphological observations (Figure 10). Furthermore, when combining these findings with the corrosion morphology of the R-UHPC surface (Figure 9), it can be found that there is a significant correlation between the corrosion severity of the rebar and the longitudinal cracks on the surface of the specimen. Specifically, when through-length longitudinal cracks become visible on the surface, the internal rebar has already developed pronounced corrosion with pitting. This strong correlation shows that monitoring surface crack characteristics on R-UHPC can serve as an effective indicator for assessing rebar corrosion damage, thereby providing a practical basis for evaluating structural corrosion severity.

## 5. Mechanical Behaviors

### 5.1. Crack Behavior and Failure Modes

**Figure 19:**
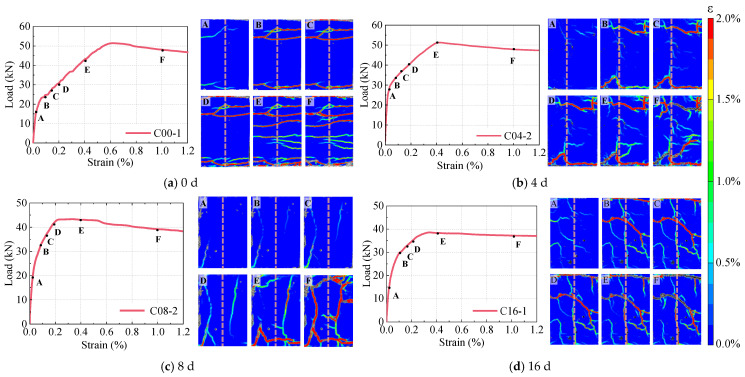
Cracking process of R-UHPC.

Through DIC analysis, this study captures the full-spectrum cracking evolution of R-UHPC under tensile loading at varying corrosion stages (Figure 19), including load-strain curves and corresponding crack patterns. Labels A–F denote different strains: initial cracking, 0.1%, 0.15%, 0.2%, 0.4%, and 1.2%. Comparing the crack development in specimens across 0, 4, 8, and 16 days of corrosion can establish the influence of corrosion severity on both crack propagation patterns and failure modes. In the uncorroded R-UHPC, initial cracking occurred at point A, with crack numbers increasing progressively from A to E. During tensile loading, predominantly horizontal cracks developed throughout the tensile segment, reaching a maximum width of 0.11 mm at point E and only 0.22 mm at peak load. These findings collectively confirm the characteristic multiple-cracking behavior and exceptional crack width control capability of uncorroded R-UHPC, a feature crucial for enhancing durability and preventing progressive deterioration in aggressive environments.

At point B, the tensile crack morphology of 4-day corroded R-UHPC specimens exhibited greater tortuosity compared to uncorroded specimens under identical strain levels, attributed to fine corrosion cracks formed due to steel fiber corrosion near the surface. These pre-existing cracks acted as stress concentration zones and governed the propagation path of tensile cracks under loading. At point E, minimal secondary crack formation became evident, with maximum crack widths reaching 0.23 mm, which is 109.1% wider than in uncorroded specimens at equivalent strain, reflecting a substantial decline in the crack resistance of specimens.

The tensile crack development in 8-day and 16-day corroded R-UHPC specimens revealed similar progression patterns, with longitudinal cracks appearing at point A and primarily expanding between points B and D without subsequent crack formation, demonstrating spatial correlation with corrosion-induced surface cracks (Figure 9). This phenomenon stems from the synergistic action of extensive rebar corrosion severely weakening the rebar–matrix bonding and extensive dissolution of steel fibers throughout the cross-section, reducing their bridging capability. Collectively, these effects led to the complete loss of R-UHPC’s characteristic multiple-cracking behavior.

**Figure 20:**
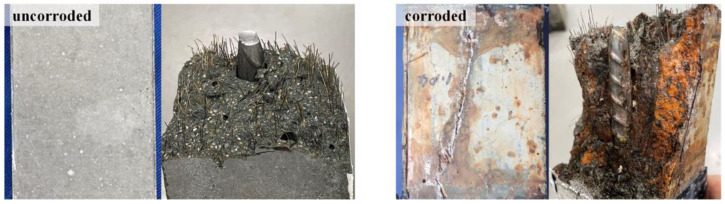
Fracture sections of R-UHPC.

The R-UHPC tensile fracture surfaces before and after corrosion (Figure 20) reveal distinct morphological differences. The uncorroded R-UHPC exhibited a relatively smooth, transversely fractured surface in deep grey coloration, characteristic of the UHPC matrix. In contrast, the post-corrosion fracture surface aligned with the orientation of longitudinal cracks and displayed extensive reddish-brown corrosion products from the steel fibers and rebar. These observations indicate that the effect of corrosion on the tensile fracture morphology of R-UHPC depends on the crack propagation through the UHPC matrix and degradation of the bonding between the rebar and matrix. Their synergistic action transitions the tensile failure mode from multiple cracking to preferential cracking along corrosion-weakened interfaces.

### 5.2. Tensile Performance

**Figure 21:**
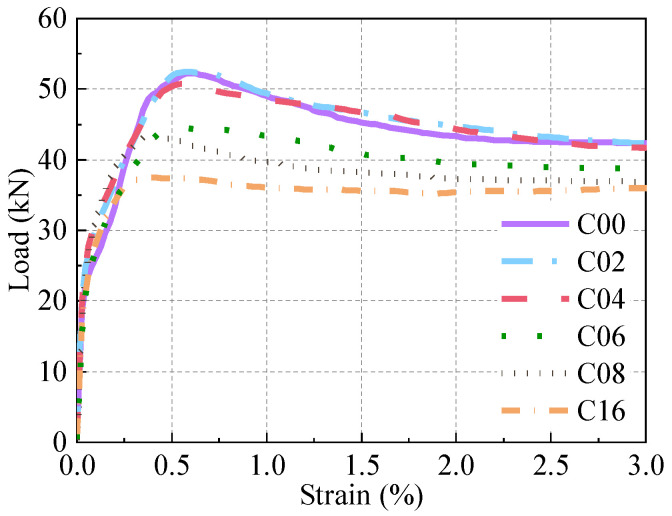
Comparison of R-UHPC with different corrosion periods.

The tensile load–strain relationships of corroded R-UHPC specimens are presented in Figure 18, and Figure 21 provides a comparative analysis of the tensile performance among specimens with varying corrosion periods within the 3.00% strain range. These experimental results reveal that R-UHPC specimens subjected to different corrosion periods exhibited consistent evolutionary patterns in their tensile response curves. Specifically, the tensile load–strain relationship exhibited linear elasticity prior to reaching 0.02% strain. Following initial cracking, the tensile load growth in R-UHPC specimens gradually slows, with specimens attaining their peak load capacities within the strain range of 0.40% to 0.60%. As the strain approaches 3.00%, the load ceases to decline and the curve exhibits a slow upward trend, corresponding to the complete fracture of the UHPC matrix components and subsequent load transfer to the rebar, which entered its strain-hardening phase and sustained loading until achieving ultimate tensile capacity.

The tensile load-bearing capacity of R-UHPC specimens exhibited a progressive decline from 53.24 kN to 37.75 kN as the corrosion duration increased from 0 to 16 days (Table 3). However, at strains exceeding 3.00%, the load–strain curve revealed consistent maximum load across all corroded specimens, maintained at approximately 43.00 kN. This load stabilization phenomenon is attributed to the complete loss of load-bearing capacity in the UHPC matrix during the advanced tensile stage, at which point the mechanical response solely reflects the rebar tensile behavior.

**Figure 22:**
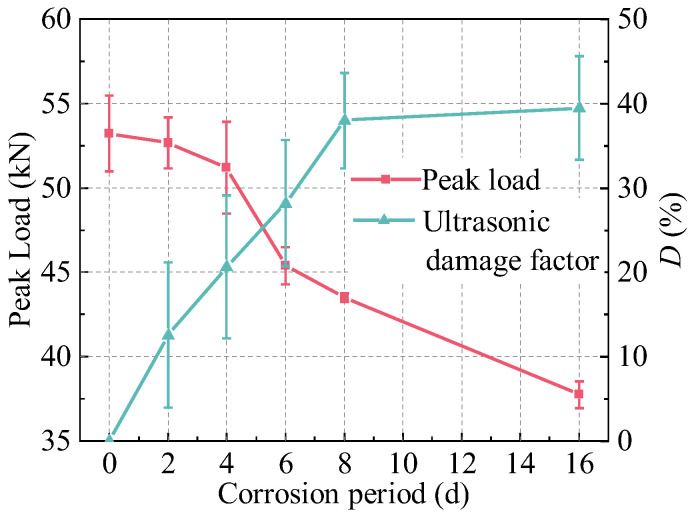
Peak load and damage factor–corrosion period curve.

Analysis of the peak load and ultrasound damage factor (*D*) variation with corrosion period in R-UHPC (Figure 22) and correlating with crack characteristics (Figure 12) reveals distinct damage progression phases. It can be seen that at 4 days of corrosion, specimens exhibited an ultrasonic damage factor of 20.6% and an average crack width of 0.21 mm, yet the peak load showed only a marginal 3.81% reduction compared to uncorroded specimens. This demonstrates that R-UHPC retains significant load-bearing capacity during initial crack formation. Subsequently, at 8 days, crack parameters escalated to 38.0% damage factor and 0.38 mm width, accompanied by an 18.31% peak load decrease, reflecting the critical threshold where complete radial crack formation and laminar crack expansion severely impair structural integrity. Beyond 8 days of corrosion, the ultrasonic damage factor stabilized due to exceeding ultrasonic detection thresholds, while mechanical degradation continued, evidenced by a further reduction in peak load to 37.75 kN, corresponding to a 29.09% reduction relative to non-corroded specimens.

A strong correlation exists between peak load reduction and progressive steel fiber loss in R-UHPC cross-sections. During the initial 4-day corrosion phase, fiber quantity remained essentially unchanged, while the peak load slightly decreased by approximately 4.00%. This reduction is attributable to minor corrosion damage resulting from the corrosion of superficial steel fibers. However, as corrosion progressed beyond 4 days, corrosive solution penetration through cracks led to accelerated fiber degradation, causing nonlinear mechanical deterioration. Specifically, 6-day corroded specimens exhibited an 11.72% reduction in fiber quantity compared to uncorroded specimens, accompanied by a sharp 11.35% drop in peak load within just 2 days, highlighting the sensitivity of mechanical performance to fiber quantity. By day 16 of corrosion, the quantity of steel fibers and the load-bearing capacity had decreased by 41.47% and 29.09%, respectively, demonstrating the critical role of fiber quantity in maintaining structural integrity and performance.

As illustrated in Figure 18, the elongation at break of R-UHPC demonstrates a trend of initial increase followed by a decrease with the extension of the corrosion period. Notably, the elongation values for 0, 4, and 16 days were 27.60%, 35.79%, and 32.82%, respectively. This trend stems from competing mechanisms: while the gradual degradation of the bonding between the rebar and the UHPC matrix and the weakening of the bridging effect of the steel fibers initially enhances ductility, the development of rebar pitting corrosion ultimately dominates the mechanical response. The formation of severe corrosion pits reduces the effective load-bearing cross-section and generates localized stress concentrations, which progressively override the initial ductility gains and cause the eventual decline in elongation.

### 5.3. Component Response of Corroded R-UHPC in Tension

**Figure 23:**
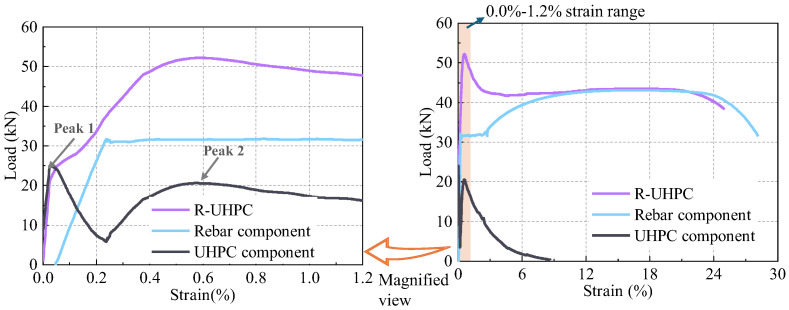
Load–strain response of components.

The total load carried by R-UHPC comprises the combined contributions of the UHPC component and the embedded rebar. Figure 23 presents the load–displacement curves for R-UHPC specimens, steel rebars, and UHPC components, where the R-UHPC load–displacement curve is derived from tensile tests, and the load–displacement curve for uncorroded rebar is obtained from steel rebar material property tests. The UHPC component curve was then calculated by subtracting the steel rebar load from the total load of the R-UHPC specimen. It should be noted that the load–displacement curve for the rebar also accounts for autogenous shrinkage effects, with prior research on similar systems indicating an adjusted initial compressive strain of 0.0473% [68].

The UHPC component exhibits a distinctive bimodal load response (Figure 23), with the first peak occurring at approximately 0.025% strain, corresponding to the initial cracking strain of R-UHPC. Following crack initiation, continuous crack development triggers load redistribution between the UHPC matrix and rebar. Due to the stiffness discrepancy, the majority of the load is progressively borne by the rebar, while the load carried by the UHPC component decreases. Upon the rebar reaching the yield stage, the crack width in the UHPC component increases with the strain. The bridging effect of steel fibers at cracks allows the specimen to sustain carrying the load. Consequently, the load on the UHPC component gradually increases to a second peak, then decreases as numerous steel fibers are pulled out at the main crack.

The characteristic peaks of UHPC components in R-UHPC at varying corrosion periods (Table 3) reveal that Peak 2 decreased significantly more than Peak 1 during corrosion, showing reductions of 68.01% and 12.79%, respectively, within 16 days. This difference occurs because Peak 2 reflects the load borne by the UHPC components during extensive steel fiber pull-out or fracture, reflecting the load-bearing capacity of UHPC components. The accelerated decline of Peak 2 after 4 days was triggered by radial crack development, allowing corrosive medium penetration, which induced extensive steel fiber corrosion and subsequent crack-induced damage. Conversely, Peak 1 represents the UHPC matrix intrinsic tensile cracking resistance, which experiences only marginal weakening since corrosion-induced cracks predominantly propagate longitudinally (Figure 9), aligning with the tensile stress direction and thus minimally compromising the cross-sectional integrity, ultimately accounting for its relatively stable performance compared to Peak 2.

**Figure 24:**
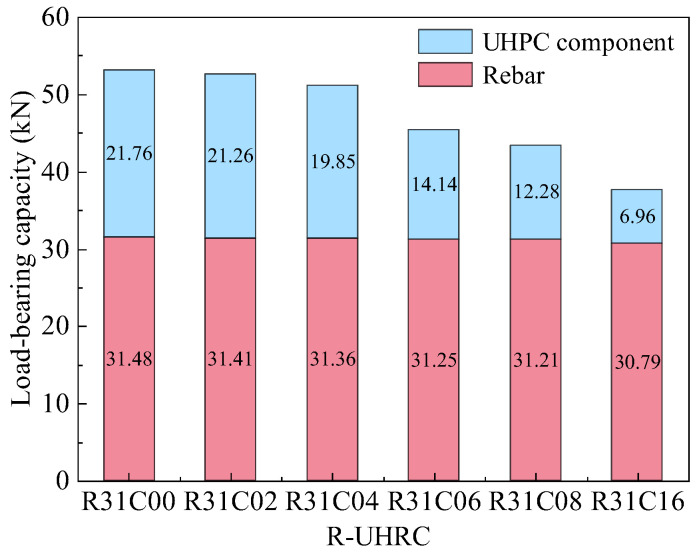
Load-bearing capacity of UHPC component and rebar based on the peak load of R-UHPC.

Analysis of the load contribution between UHPC components and steel rebar in the peak load of R-UHPC (Figure 24) reveals that, within the first 4 days of corrosion, the load carried by the steel fibers decreased from 21.76 kN to 19.85 kN and the load carried by the rebar dropped from 31.48 kN to 31.36 kN. Particularly, their proportion of the R-UHPC peak load changed by no more than 2%, indicating minimal early-stage corrosion effects on bearing capacity. As corrosion progresses, the load carried by UHPC components rapidly decreases, whereas the reduction in load borne by the steel rebar remains marginal. Specifically, within 16 days of corrosion, the actual load borne by the rebar in R-UHPC decreased by 0.69 kN, while its share of R-UHPC peak load increased from 59% to 82%. In contrast, the load borne by the UHPC component decreased from 21.76 kN to 6.96 kN, accounting for 96% of the total reduction in R-UHPC peak load. This indicates that the decline in the load-bearing capacity of the UHPC component is the primary factor driving the reduction in R-UHPC peak load during this 16-day corrosion period. The underlying mechanism can be attributed to the rebar-induced resistivity reduction, which significantly accelerated the electrochemical corrosion process in R-UHPC, leading to corrosion-induced reductions in steel fiber quantity and cracking damage in the UHPC that caused a substantial decline in the load-bearing capacity of the UHPC component.

## 6. Conclusions

Accelerated corrosion testing and subsequent direct tension experiments evaluated the corrosion performance and post-corrosion tensile behavior of R-UHPC, examining corrosion progression, corrosion-induced cracking patterns, tensile crack development, and mechanical response under tension. Crack width measurement and ultrasonic nondestructive testing were employed to monitor cracking damage. The following conclusions are reached:

1. The embedding of rebar in UHPC leads to the development of F-R conductive pathways, altering corrosion-induced cracking behavior. Beyond the laminar cracks induced by corrosion along F-F pathways, corrosion at F-R pathways generates radial cracks that direct corrosive solution to the rebar, accelerating the electrochemical corrosion process of the specimen.
2. Corrosion progressively degrades the tensile crack resistance of R-UHPC. As corrosion advances, the bridging effect of steel fibers deteriorates, and the debonding between rebar and matrix intensifies. These combined effects lead to the gradual loss of R-UHPC’s characteristic multiple-cracking behavior, critically undermining its durability in aggressive environments.
3. R-UHPC load-bearing capacity exhibited a transition from gradual to accelerated deterioration phases with corrosion period, aligning with steel fibers reduction temporally. In the initial 4 days of corrosion, only surface-level corrosion and cracking occurred, accompanied by minimal steel fiber loss and a marginal 3.8% reduction in load-bearing capacity. As corrosion progressed, steel fiber content decreased by 22.6% and 41.5% after 8 and 16 days, respectively, accompanied by corresponding bearing capacity reductions of 18.3% and 29.1%.
4. The ultrasonic damage factor and the corrosion crack width exhibit approximately linear growth trends before reaching critical damage thresholds, which serve as the indicators of internal and surface corrosion damage in R-UHPC, providing a cost-effective approach to estimate the residual load-bearing capacity of in-service structures non-destructively.

## Figures and Tables

**Table 1:** Mechanical properties of UHPC.

Fiber Volume Fraction (vol%)	Tensile Strength *f*_t_ (MPa)	Compressive Strength *f*_c_ (MPa)	Elastic Modulus *E* (GPa)
2.0	10.9	156.1	46.1

**Table 2:** Mechanical properties of steel rebar.

Steel Rebar	Diameter *d* (mm)	Elastic Modulus *E* (GPa)	Yield Strength *f*_y_ (MPa)	Ultimate Strength *f*_u_ (MPa)
HRB400	10	214	401	549

**Table 3:** Specimen details.

Group	Corrosion Period (d)	Peak Load (kN)	Peak 1 (kN)	Peak 2 (kN)
C00	0	53.24	25.02	21.76
C02	2	52.67	25.59	21.26
C04	4	51.21	25.57	19.85
C06	6	45.39	23.21	14.14
C08	8	43.49	24.41	12.28
C16	16	37.75	21.82	6.96

## Data Availability

The original contributions presented in this study are included in the article. Further inquiries can be directed to the corresponding author.
